# miRNA-135a promotes breast cancer cell migration and invasion by targeting *HOXA10*

**DOI:** 10.1186/1471-2407-12-111

**Published:** 2012-03-23

**Authors:** Yating Chen, Jin Zhang, Huijun Wang, Jiayi Zhao, Cheng Xu, Yingying Du, Xin Luo, Fengyun Zheng, Rui Liu, Hongwei Zhang, Duan Ma

**Affiliations:** 1 Key Laboratory of Molecular Medicine, Ministry of Education, Shanghai Medical College, Fudan University, Shanghai 200032, China; 2 Children"s Hospital of Fudan University, Shanghai 201102, China; 3 Department of Surgery, Zhongshan Hospital, Fudan University, Shanghai 200032, China; 4 Institutes of Biomedical Science, Fudan University, Shanghai 200032, China

## Abstract

**Background:**

miRNAs are a group of small RNA molecules regulating target genes by inducing mRNA degradation or translational repression. Aberrant expression of miRNAs correlates with various cancers. Although miR-135a has been implicated in several other cancers, its role in breast cancer is unknown. *HOXA10 *however, is associated with multiple cancer types and was recently shown to induce p53 expression in breast cancer cells and reduce their invasive ability. Because *HOXA10 *is a confirmed miR-135a target in more than one tissue, we examined miR-135a levels in relation to breast cancer phenotypes to determine if miR-135a plays role in this cancer type.

**Methods:**

Expression levels of miR-135a in tissues and cells were determined by poly (A)-RT PCR. The effect of miR-135a on proliferation was evaluated by CCK8 assay, cell migration and invasion were evaluated by transwell migration and invasion assays, and target protein expression was determined by western blotting. GFP and luciferase reporter plasmids were constructed to confirm the action of miR-135a on downstream target genes including *HOXA10*. Results are reported as means ± S.D. and differences were tested for significance using 2-sided Student"s t-test.

**Results:**

Here we report that miR-135a was highly expressed in metastatic breast tumors. We found that the expression of miR-135a was required for the migration and invasion of breast cancer cells, but not their proliferation. *HOXA10*, which encodes a transcription factor required for embryonic development and is a metastasis suppressor in breast cancer, was shown to be a direct target of miR-135a in breast cancer cells. Our analysis showed that miR-135a suppressed the expression of *HOXA10 *both at the mRNA and protein level, and its ability to promote cellular migration and invasion was partially reversed by overexpression of *HOXA10*.

**Conclusions:**

In summary, our results indicate that miR-135a is an onco-miRNA that can promote breast cancer cell migration and invasion. *HOXA10 *is a target gene for miR-135a in breast cancer cells and overexpression of *HOXA10 *can partially reverse the miR-135a invasive phenotype.

## Background

Micro RNAs (miRNAs) are small non-coding, cellular RNAs (17-27 bp) that post-transcriptionally regulate gene expression by inducing the degradation or translational repression of target mRNAs. The discovery of miRNAs and their mode of action has revealed an entirely new level of gene regulation. miRNAs must be assembled into a complex termed the RNA induced silencing complex (RISC) in order to regulate expression of their mRNA targets. Once assembled they act by binding to the 3'untranslated region (3'-UTR) and inducing degradation or transcriptional repression [1]. An individual miRNA is capable of regulating hundreds of distinct mRNAs, and more than 1,000 human miRNAs have been identified that could potentially modulate close to one-third of the coding genes in human genome [2].

Aberrant expression of miRNAs has been correlated with various human diseases including cancers. miRNAs have been identified which have oncogenic or tumor suppressor properties because the target genes they regulate are oncogenes or tumor suppressor genes. The abnormal expression profiles of miRNAs have been examined in many different cancers including breast cancer [3,4] and their roles in the proliferation, apoptosis, invasion/metastasis and angiogenesis of normal and cancer cells are being investigated aggressively [5-9].

The function of the miRNA miR-135a is currently under investigation in our laboratory. Processes known to be under the control of miR-135a include megakaryocytopoiesis[10], bone and muscle development, hypertension, colorectal cancer through its target gene *Adenomatous Polyposis Coli (APC)*[11-13], epithelial ovarian cancer and endometriosis through its target gene *HOXA10*[14], portal vein tumor thrombus through its target gene *metastasis suppressor 1 *and Hodgkin disease and gastric cancer through its target JAK2[15,16]. At present however, its role in breast cancer is unknown.

Normal development and tumorigenesis both depend on shifts in the delicate balance between cell growth and differentiation. Altered expression of genes that are involved in the transcriptional control of developmental pathways often contribute significantly to oncogenesis because cancer can arise from the misappropriation of signalling pathways normally used to control cell fate [17]. The *Homeobox *genes encode transcription factors that are critical for the proper placement of segment structures during embryonic development. Analysis of numerous tumors have revealed that the expression of specific *HOX *genes is often increased or decreased, indicating that they can influence tumor suppression or tumor development, invasion, and metastasis [18]. Several *HOX *genes have been shown to be partially regulated by miRNAs [6,19-21], indicating the potential for oncogenic pathways that begin with miRNA dysregulation, leading to altered *HOX *gene expression and ultimately tumorigenesis or tumor suppression. The *HOXA10 *gene is a regulator of embryonic morphogenesis and differentiation and is aberrantly expressed in several types of cancers [22-29]. Recently it was reported to induce p53 expression in breast cancer cells and to reduce their invasiveness [30]. Of specific interest, *HOXA10 *has been shown to be under the control of miR-135a in endometrial and epithelial cancer tissues [14] which led us to ask if miR-135a might also be associated with breast cancer.

In this study, we found that miR-135a levels are elevated in breast cancer with metastasis. By manipulating the expression level of miR-135a *in vitro*, we showed that miR-135a could promote the migration and invasiveness of breast cancer cells. We used bioinformatic tools and the literature to identify *HOXA10 *as a miR-135a target gene candidate and verified it is directly regulated by miR-135a in breast cancer cells. We found that over expression of *HOXA10 *could partially reduce the invasive property mediated by elevated miR-135a levels in the breast cancer cell line BT549.

## Methods

### Cell culture and tissue samples

All cell lines were obtained from the American Type Culture Collection. HEK293, human breast cancer cell lines BT549, SKBr3 and MDA-MB-231 were cultured in Dulbcco"s Modifed Eagle Medium (Gibco, Grand Island, NY, USA). All cell lines were incubated at 37°C in 5% CO2. Patients samples are collected from Zhongshan Hospital, Fudan University. This research was taken under consent of all patients for the use of their samples. This program is got approval by the Institute of Biomedical Sciences ethics committee of Fudan University. Ten Benign patient diagnosed as adenosis or fibroadenoma, fifteen invasive breast cancer as malignant samples whose ER(-), PR(-) and CerbB-2 (-) markers are all negative and all have lymph node metastasis. Fresh tissue samples were harvested from patients, and preserved at -80°C.

### Detection of mRNAs and miRNAs

Total RNA from cells and human tissue samples were extracted using Trizol (Invitrogen) according to the manufacturer"s instructions and 2 μg of each total RNA sample was aliquoted to synthesize cDNA using the Reverse Transcription System (Promega). For mRNA detection and expression, *HOXA10 *and *β-actin *were analyzed by RT-PCR or qRT-PCR. All qRT-PCR products were amplified using a SYBR green PCR Master Mix kit (Qiagen) according to the manufacturer"s instructions on the ABI Prism 7,500 Detection System (Applied Biosystems). For quantification of *HOXA10 *mRNA in transfected cells, *β-actin *was used as the internal control. Levels of mRNA were quantified based on the ratio of *HOXA10 *mRNA/β-actin mRNA using the 2 - ΔΔCt method where ΔΔCt = ΔCtexp - ΔCtnc = (Ctexp-target - Ctexp-actin) - (Ctnc-target - Ctnc-actin), in which "exp" represents the experimental group, "nc" the negative control group, and "target" the gene of interest.

For miRNA detection, we employed Poly (A) RT-PCR method using specific forward primers and a universal reverse primer complementary to an anchor primer as previously described [31]. The anchor RT primer was used as the template for the negative control and the U6 small nuclear RNA was used as the control to determine relative miRNA expression. Levels of miR-135a in human tissue samples were quantified and normalized to 18S rRNA using the 2 - ΔΔCt method formula as described above. The PCR profile was one cycle of 95°C for 5 min, then 40 cycles of 95°C for 5 s and 60°C for 50 s. The primers used for detection are listed in Table 1.

**Table 1:** Primers for detection of *HOXA10 *mRNA and miR-135a by RT-PCR

Primers	Sequence
*miR-135 RF*	cgcgtctatggctttttattccta
*anchor RT primer*	cgactcgatccagtctcagggtccgaggtattcgatcgagtcgcactttttttttttt
*Universal rev primer*	ccagtctcagggtccgaggtattc
*U6F*	ctcgcttcggcagcaca
*U6T*	aacgcttcacgaatttgcgt
*HOXA10 RT F*	ctggtcccctccctctgtc
*HOXA10 RT T*	acaacaaataaaccagcaccaag
*β-actin F*	ccttcctgggcatggagtcct
*β-actin T*	aatctcatcttgttttctgcg

### Plasmid construction and transfection

A DNA fragment encoding the miR-135a pre-miRNA was amplified by PCR from HEK293 genomic DNA and cloned into a modified pSilencer 4.1-CMVneo vector (Ambion) as previously described [31]. Positive clones were identified by PCR screening and DNA sequencing. Using genomic similarly, 3"-UTRs from the predicted mir-135a target genes *HOXB7, APC *and *HOXA10 *were PCR amplified from HEK293 DNA and cloned into the XbaI site immediately downstream of the stop codon in the pGL3-promoter vector (Promega). To produce mutant *HOXA10 *3'-UTR pGL3-reporter plasmids, the predicted miR-135a binding sites were replaced with 18 bp-long fragments (Table 2) by overlapping PCR. Fragments of *HOXA10 *containing 3' UTR regions were cloned from a *HOXA10 *cDNA into pEGFP-C1 for pEGFP-*HOXA10 *plasmid construction. The *HOXA10 *expression vector pcDNA3-hisC-*HOXA10 *and vector pcDNA-*HOXA10*-de-3'UTR were kindly provided by Dr Herring (Indiana University, USA). The miR-135a anti-sense oligonucleotide inhibitor (miR-135a inhibitor) and a mismatched sequence (N.C) were purchased from GenePharma (Shanghai, China). *HOXA10 *and Control siRNAs were from Santa Cruz (sc-38685, sc-37007). The primers used were listed in Table 2. All plasmid DNAs used for transfection were prepared using a Qiagen DNA Miniprep kit following the manufacturer"s instructions. Cells were transiently transfected with vectors or anti-miR inhibitor using Lipofectamine 2000 from Invitrogen (CA, USA) according to the manufacturer"s instructions.

**Table 2:** Sequences of PCR primers for plasmid construction

Primers	Sequence
*HOXA10 UTR F*	ccTCTAGActggtcccctccctctgtc
*HOXA10 UTR T*	ccTCTAGAgatagggagaattgtggtgtgc
*HOXB7 UTR F*	ggTCTAGAgggcagaggaagagacatgag
*HOXB7 UTR T*	ggTCTAGAgggttagtccagacccacag
*APC UTR F*	ggTCTAGAttaaaagagaggaagaatgaaactaag
*APC UTR T*	ggTCTAGAgcatgtatctccattgtttatgg
*muHOXA10 F*	TCTTGGATCCTTCAAGTCtcatgctaaaattctatagagatag
*muHOXA10 T*	GACTTGAAGGATCCAAGAacaacaaataaaccagcaccaag

### *In vitro *luciferase assay

HEK293 cells (1 × 10^5^) were seeded into a 96-well plate and co-transfected with 5 ng of internal control vector pRL-renilla (Promega), 50 ng of a pGL3-promoter reporter with either the *HOXA10*, mutant *HOXA10, HOXB7 *or *APC *3'-UTR and 150 ng of the pSilencer-135a (pS-135a) or pSilencer-4.1-CMV-negative (pS-negative) vector. Forty eight hours after transfection, the firefly and Renilla luciferase activities were assayed using the Dual-Glo Luciferase assay system from Promega according to the manufacturer"s protocol. For each sample firefly luciferase activity was normalized to the Renilla luciferase activity value.

### Western blotting

Whole-cell extracts were prepared using RIPA lysis buffer and SDS-PAGE followed by Western blotting were performed as previously described [31] and signals were visualized with Super Signal West Femto chemiluminescent substrate. For each condition, samples were analyzed three times independently. The primary antibodies used were from Santa Cruz Biotechnologies for *HOXA10 *(sc-28602), Sigma for β-actin (A7441), and Cell Signaling for GFP (#2956). The peroxidase-conjugated anti-mouse or anti-rabbit IgG secondary antibodies were from Santa Cruz Biotechnology.

### Cell proliferation assay

MDA-MB-231 or BT549 (1 × 10^4 ^cells per well) were plated into 96-well plates in triplicate prior to pSilencer 4.1-CMV-135a or pS-negative transfection and then cultured for approximately 16 h. Cell proliferation was assessed 72 h after transfection using CCK8 (Dojindo, Tokyo, Japan) according to the manufacturer"s instructions.

### Migration and invasion assay

Cell migration and invasive ability was examined using a 24-well transwell plate with 8 mm pore polycarbonate membrane inserts, according to the manufacturer"s protocol (Corning, NY, USA). The matrigel (14.8 μg/ml) employed for the invasion assays was applied to the upper surface of the membranes. Forty eight hours after transfection, 5 × 10^4 ^cells per well were seeded into the top chamber in serum-free media and this was replaced with complete growth media for 12 h. Cells that migrated or invaded through surface of the membrane were fixed with methanol and stained with hematoxylin. Migrating or invasive cells from three random microscope fields per filter were selected for cell counting.

### Bioinformatics prediction and statistics analysis

We computationally screened proper targets for miR-135a by miRNAMap http://mirnamap.mbc.nctu.edu.tw/. Statistical analysis for miR-135a expression in tissues was performed using a Jonckheere-Terpstra exact test for trend to compare the distribution of expression levels (high, medium, low, none) across breast tumors and benign tissues. A Bonferroni adjustment was applied to the *p *values for the pair-wise comparisons. Results were delineated as means ± S.D., differences were tested for significance using 2-sided Student"s *t*-test.

## Results

### miR-135a levels were elevated in human metastatic breast tumors

**Figure 1:**
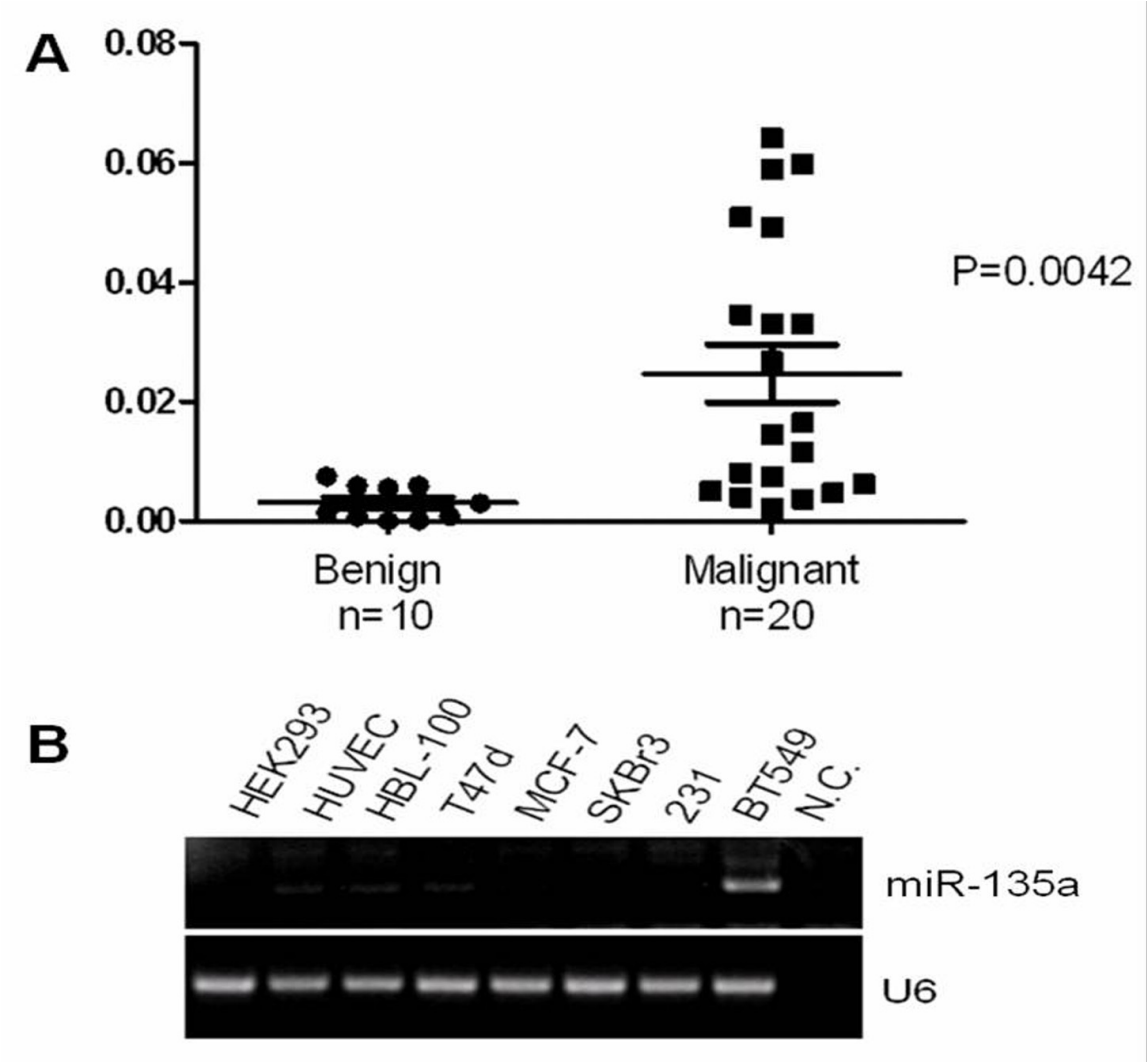
**Expression of miR-135a was elevated in human breast tumors with metastasis**. (A) miR-135a levels were detected in 30 human breast samples by SYBR Green qRT-PCR with 18S rRNA used as a loading control. For benign tumors (adenosis or fibroadenoma) n = 10 and for „triple negative" invasive malignant tumors (ER (-), PR (-) and CerbB-2 (-) markers are all negative and all have lymph node metastasis positive) n = 20. *p*-values were obtained using a 2-sided Student"s *t*-test. (B) Endogenous miR-135a detected in breast cancer cell lines by poly-A RT-PCR. U6 was used as the control.

Expression levels of miR-135a were detected from 30 human primary breast tumor samples using poly (A)-RT-PCR, as previously described [31]. The patient samples in our study we choose have very similar clinico-pathological features. We choose benign breast samples diagnosed as adenosis or fibroadenoma, and choose "triple negative" invasive breast cancer as malignant samples whose ER(-), PR(-) and CerbB-2 (-) markers are all negative and have lymph node metastasis, the prognosis of these patients are relative bad. We found that the average miR-135a level was higher in the „triple negative" malignant invasive tumors than that in the benign tumors (Figure 1A). The expression levels of miR-135a in breast cancer cell lines that included T47d, MCF-7, SKBr3, MDA-MB-231, and BT549 were also examined. We found that miR-135a was up-regulated in breast cancer cell line BT549 which has a highly invasive phenotype (Figure 1B).

### miR-135a increased breast cancer cells migration and invasion properties

**Figure 2:**
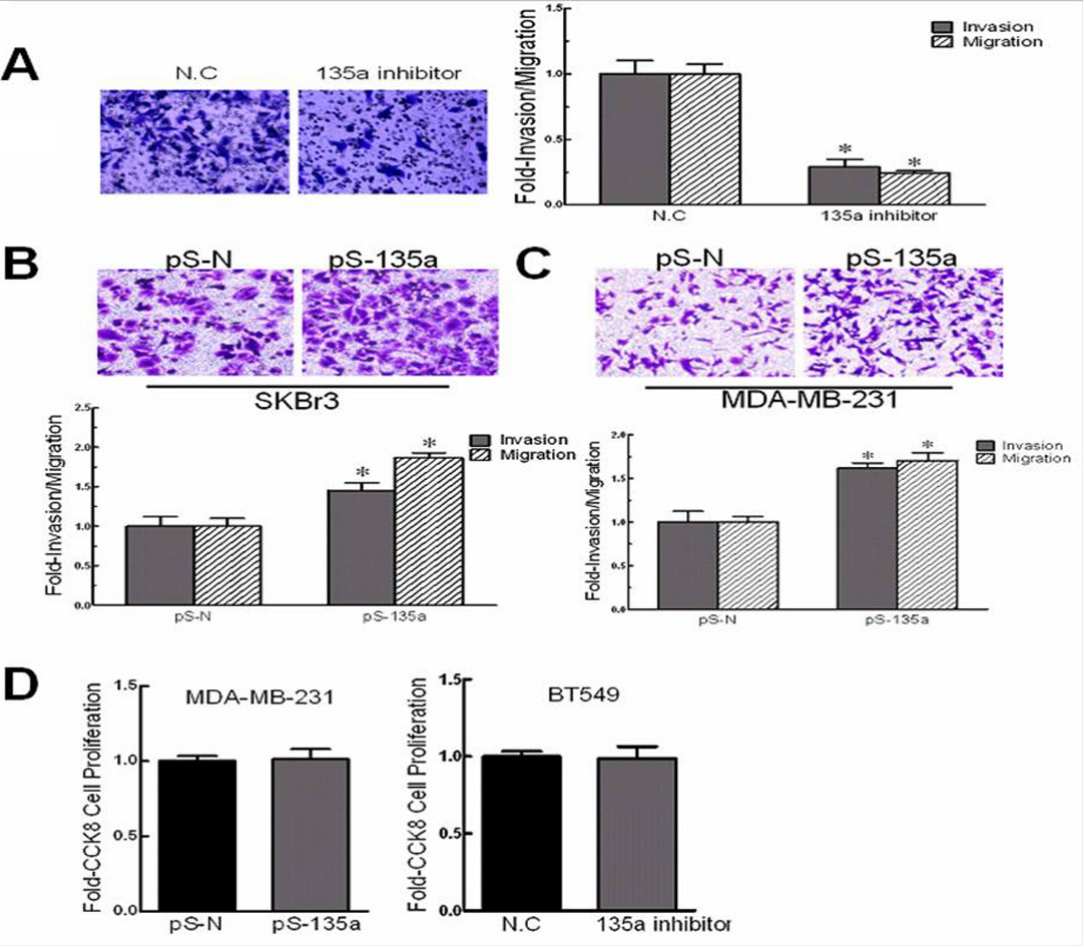
**miR-135a increased breast cancer cells migration and invasion properties**. (A) Migration and invasion after transfection of BT549 cells with a miR-135a inhibitor or no inhibitor (N.C.). Images of invasive BT549 cells (left panel). (B) Migration and invasion assays after transfection of SKBr3 cells with pSilencer-miR-135a (pS-135a) or pSilencer4.1CMV-negative (pS-N). Images of invasive SKBr3 cells (top panel). (C) Migration and invasion assays after transfection of MDA-MB-231 cells with pS-135a or pS-N. Images of invasive MDA-MB-231 cells (top panel). (D) Cell proliferation of MDA-MB-231 cells transfected with pS-135a or pS-N, and BT549 cells transfected with the miR-135a inhibitor or negative control (N.C). n = 3, *, *p *< 0.05, significantly decreased migration or invasion.

Cell invasion is a significant aspect of cancer progression which involves the migration of tumor cells into contiguous tissues and the dissolution of extracellular matrix proteins [7]. Because we found that miR-135a was highly expressed in the „triple negative" malignant invasive human tumors and the BT549 tumor line, which has a highly invasive phenotype, we asked if miR-135a overexpression contributed to these phenotypes. We transfected BT549 cells with miR-135a inhibitor (2'-O-methylated oligonucleotides) to block miR-135a action and performed a migration assay with the cells 48 h after transfection. The miR-135a inhibitor impeded the migration of BT549 cells from serum-free medium to serum-containing medium, reducing it by 2-fold (Figure 2A). In addition, the invasion of miR-135a inhibitor transfected BT549 cells was also significantly compromised (Figure 2A). These effects appear to be specifically attributable to the miR-135a inhibitor blocking the biological action of miR-135a because the NC mismatched control inhibitor failed to affect either migration or invasion.

Because the inhibition of miR-135a reduced BT549 cell migration and invasion, we assessed the effect of miR-135a overexpression in cell lines SKBr3 and MCF-7 using a pSilencer-135a vector (see Methods). The forced expression of miR-135a resulted in a ~2-fold increase of SKBr3 cell migration (Figure 2B). Consistent with this result, the invasive activity of SKBr3 cells across an extracellular matrix also increased. But no change on migration and invasive activity was observed in MCF-7 cells (data not shown). To confirm that the ability of miR-135a to promote cell migration and invasion was not restricted to SKBr3 cells, we tested a third human breast cancer cell line, MDA-MB-231. The migration and invasive activity of these cells was also enhanced by overexpressing miR-135a (Figure 2C), suggesting that miR-135a function can promote migration and invasion in breast cancer cells.

We also tested if miR-135a could affect cancer cell proliferation by overexpression, down-regulation or both. The results demonstrated that altered miR-135a expression neither increased nor decreased cell growth compared to control cells (Figure 2D). In summary, our data indicates that miR-135a can increase breast cancer cell migration and invasiveness, but has no obvious effect on cell proliferation.

### *HOXA10 *is a direct target of miRNA-135a in breast cancer cells

**Figure 3:**
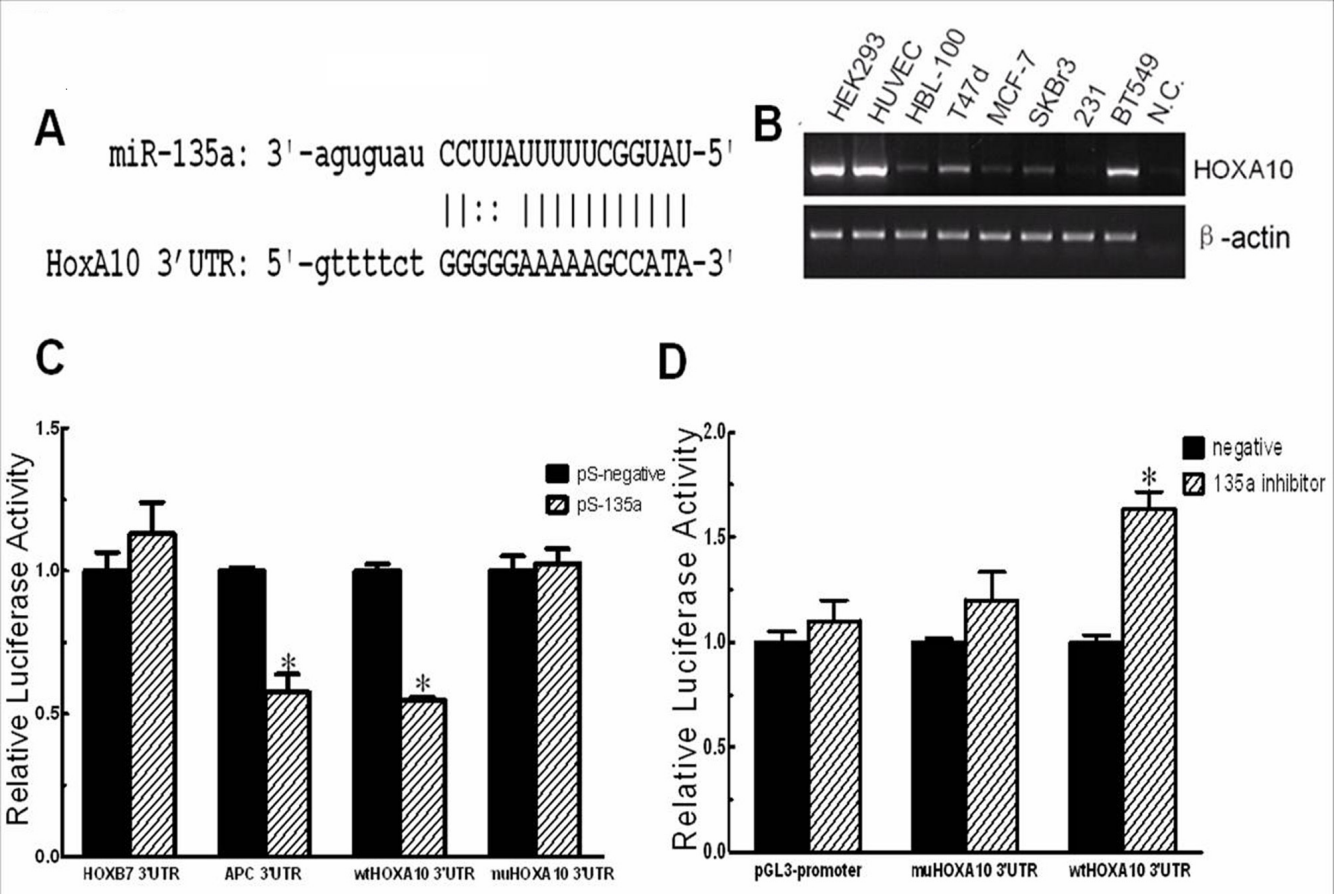
***HOXA10 *as a target of miR-135a**. (A) Online prediction of miR-135a potential binding sites on *HOXA10 *3'-UTR. The nucleotide sequence illustrates the predicted base-pairing between miR-135a and the *HOXA10 *3'-UTR. (B) The endogenous mRNA expression of *HOXA10 *was detected by RT-PCR in breast cancer cell lines with *β-actin *used as an expression control. (C) *In vitro *luciferase reporter assay in HEK293 cells. pS-135a or pS-N was co-transfected with the pGL3-3'-UTR reporter plasmids containing the HOXB7 3'-UTR, APC 3'-UTR, wide-type (wt) *HOXA10 *3'-UTR, and mutant(mu) *HOXA10 *3'-UTR. The 3'-UTR HOXB7 reporter plasmid was the negative control, and the APC reporter plasmid was the positive control. (D) *In vitro *luciferase reporter assays in BT549 cells. Negative (pGL3-promoter), wild-type (wt) and mutated (mu) 3'-UTR reporter plasmids of *HOXA10 *were cotransfected with an anti-sense inhibitor of miR-135a (135a inhibitor) or no inhibitor into BT549 cells, which express endogenous miR-135a. n = 3, *, *p *< 0.05, significantly decreased/increased activity.

We used miRNAMap online http://mirnamap.mbc.nctu.edu.tw/ to predict the targets of miR-135a, we chose *HOXA10 *for further analysis because of its relatively high prediction score, and complementary structure with miRNA-135a (Figure 3A). To verify *HOXA10 *is a real target of miR-135a in breast cancer, we first tested if *HOXA10 *is co-expressed with this miRNA. *HOXA10 *mRNA levels were measured by RT-PCR in HEK293, HUVEC, HBL-100 and several breast cancer cell lines. We found *HOXA10 *was indeed expressed in most breast cancer cell lines except MDA-MB-231 (Figure 3B). We next tested if miR-135a could repress *HOXA10 *using an in vitro luciferase reporter assay. Plasmid DNA of pGL3-promoter based 3'-UTR reporter constructs were co-transfected with pSilencer4.1CMV-135a (pS-135a) or the pSilencer4.1CMV-negative control (pS-negative or pS-N). As a positive control for miR-135a activity we used a, pGL3-*APC *3'-UTR construct and used a pGL3- *HOXB7 *3'-UTR construct as a negative control. Coexpression of the *HOXA10 *luciferase reporter with miR-135a greatly reduced the luciferase activity. And this effect was largely eliminated when the sites in *HOXA10 *3'-UTR targeted by miR-135a were mutated (Figure 3C). Conversely, co-transfected miR-135a inhibitor with pGL3- *HOXA10 *3'-UTR in BT549 cells, which express miRNA-135a endogenously, increased luciferase activity (Figure 3D). These observations suggested that *HOXA10 *is a direct target of miRNA-135a.

**Figure 4:**
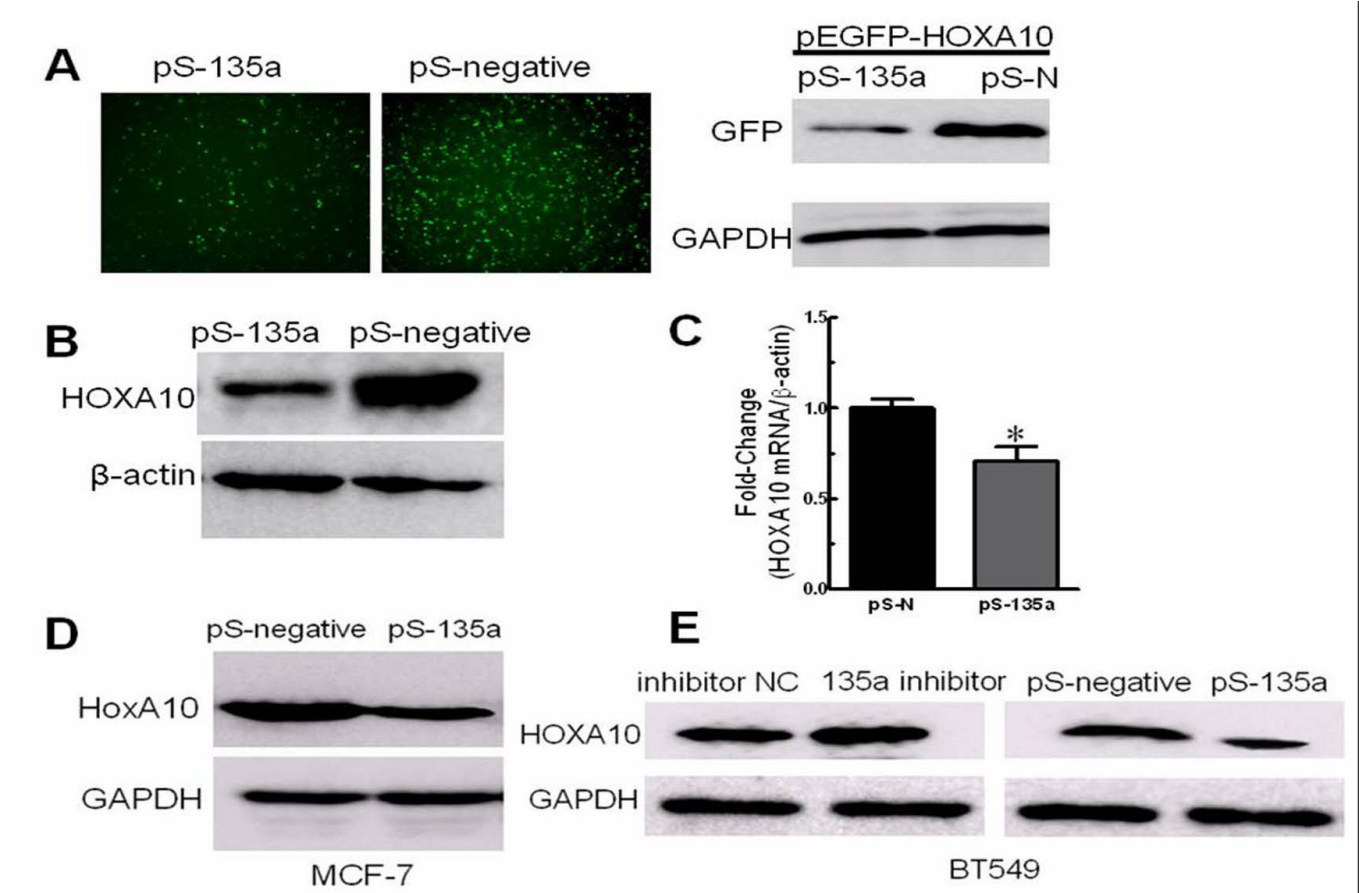
**miR-135a regulation of *HOXA10 *expression**. (A) Images of GFP-labeled HEK293 cells (left panel). Western blot of GFP protein in GFP-labeled HEK293 cells (right panel). Anti-GAPDH antibody was used as a loading control (bottom panel). The HEK293 cells were co-transfected with plasmids pEGFP-*HOXA10 *and pSilencer-miR-135a (pS-135a) or pEGFP-*HOXA10 *and pSilencer4.1CMV-negative (pS-N) respectively. (B) Western blot of *HOXA10 *protein in HEK293 cells transfected with pS-135a or pS-N. Anti-actin antibody was used as a loading control (bottom panel). (C) *HOXA10 *mRNA expression in pS-135a or negative transfected HEK293 cells detected by SYBR Green qRT-PCR. *β-actin *was used as a loading control. (D) Western blot of *HOXA10 *protein in MCF-7 cells transfected with pS-135a or pS-N. Anti-GAPDH antibody was used as a loading control (bottom panel). (E) Western blot of *HOXA10 *protein in BT549 cells transfected with inhibitor control (inhibitor NC) or 135a inhibitor, pS-135a or pS-N. Anti-GAPDH antibody was used as a loading control (bottom panel). n = 3, *, *p *< 0.05, pS-135a significantly decreased mRNA levels (normalized to *β-actin *mRNA) compared with cells transfected with pS-N.

Next, we used HEK293 cells because they expressed *HOXA10 *endogenously (Figure 3B), but expressed minimal level of miR-135a (Figure 1B), providing a good cell model system to determine the relationship between miR-135a and *HOXA10 *expression in vitro. Co-expression of pEGFP-*HOXA10 *with the pSilencer-135a in HEK293 cells led to a decreased expression of the GFP-*HOXA10 *fusion protein compared to the negative control (Figure 4A). In addition, introducing miR-135a by transfection of pS-135a into HEK293 cells reduced the endogenous *HOXA10 *protein expression (Figure 4B). We also detected a decrease of endogenous *HOXA10 *mRNA by real-time RT-PCR 48 h after transfecting HEK293 cells with pS-135a (Figure 4C). So we believe that miRNA-135a regulates *HOXA10 *expression at both protein and mRNA levels. In breast cancer cell lines, expressing miR-135a by transfection of pS-135a reduced the endogenous *HOXA10 *protein expression in MCF-7 cells (Figure 4D) and BT549 cells (Figure 4E). Besides, inhibition of miR-135a in BT549 cells increased *HOXA10 *protein expression (Figure 4E). These findings strongly indicate that *HOXA10 *is a target of miR-135a in breast cancer cells.

### Overexpression of *HOXA10 *partially reversed the invasive property of BT549 cells caused by miR-135a

**Figure 5:**
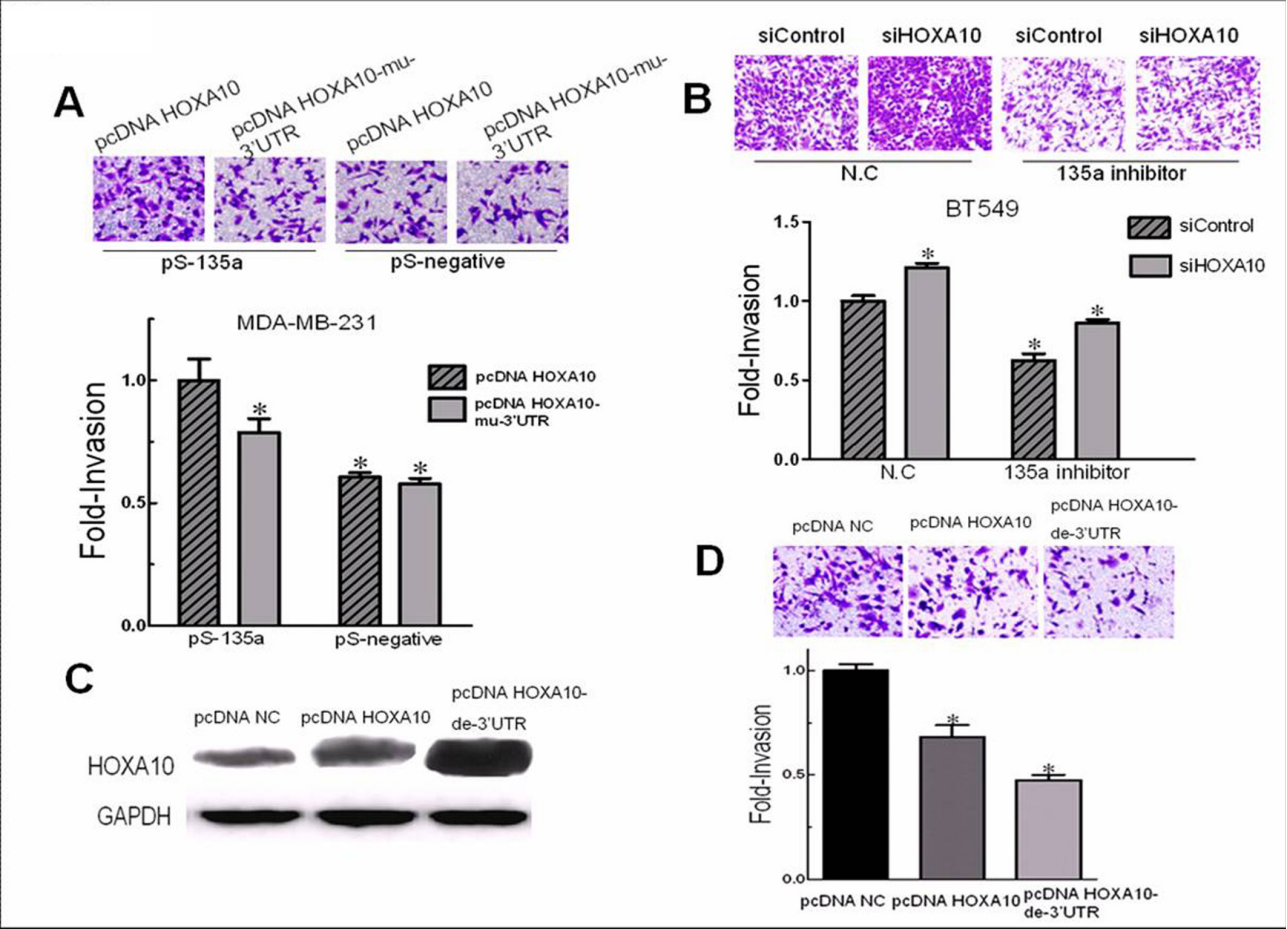
**Expression of *HOXA10 *reversed cell invasive property caused by miR-135a in BT549 cells**. (A) Invasion assay of MDA-MB-231 cells 48 h after cotransfecting pS-135a or pS-negative and pcDNA *HOXA10 *contains a full-length *HOXA10 *cDNA, or pcDNA *HOXA10*-mu-3'UTR, mutated in the miR-135a binding site in the 3'-UTR. Images of invasive MDA-MB-231 cells (top panel). (B) Invasion assay of BT549 cells 48 h after cotransfecting 135a inhibitor or inhibitor N.C and *HOXA10 *siRNA (si*HOXA10*) or Control siRNA (siControl). Images of invasive BT549 cells (top panel). (C) Western blot of *HOXA10 *protein in BT549 cells 48 h after expressing *HOXA10 *(full-length or 3'-UTR region deleted) by transfection. Anti-GAPDH antibody was used as a loading control (bottom panel). (D) Invasion assay of BT549 cells 48 h after transfection. pcDNA NC was used as negative control, pcDNA *HOXA10 *contains a full-length *HOXA10 *cDNA including its 3' UTR, and pcDNA *HOXA10*-de-3'UTR contains the *HOXA10 *coding sequence with the 3'-UTR region deleted. Images of invasive BT549 cells (top panel). n = 3, *, *p *< 0.05, significantly decreased migration or invasion.

To test whether *HOXA10 *regulation contributed to effect of miR-135a on migration and invasion, we cotransfected miR-135a (or negative control) and a vector pcDNA-*HOXA10*-mu-3'UTR containing HOXA10, mutated in the miR-135a binding site in the 3'-UTR into MDA-MB-231 cells, and found that HOXA10"s role of suppressing cell invasiveness was not limited by miR-135a when the sites in HOXA10 3'-UTR targeted by miR-135a were mutated. Besides, overexpressing *HOXA10 *with either pcDNA-*HOXA10 *or pcDNA-*HOXA10*-mu-3'UTR vector decreased invasiveness of MDA-MB-231 cells (Figure 5A). Conversely, downexpressing *HOXA10 *increased the invasive property of BT549 cells (Figure 5B). In addition to MDA-MD-231, the similar rescue experiment was carried out in BT549 cells. Compared with miR-135a knockdown alone, double knockdown of miR-135 and *HOXA10 *in BT549 could partially rescue the decreased invasive property caused by 135a inhibitor (Figure 5B).

We then transfected BT549 cells with *HOXA10*-expressing vectors that included a 3'-UTR deleted gene (pcDNA HOXA10-de-3'UTR), a full-length *HOXA10 *positive control (pcDNA HOXA10) and a negative control (pcDNA negative). Western blot analysis showed that the level of the HOXA10 protein expressed from the construct lacking the HOXA10 3'-UTR was the highest compared to the cells transfected with vectors containing the full-length *HOXA10 *cDNA or the negative control (Figure 5C). Invasion assays on BT549 cells showed that overexpression of *HOXA10 *reversed the effect of miR-135a on invasion by at least 40% (Figure 5D). However, in the BT549 cells which express high levels of endogenous miR-135a over-expression of *HOXA10 *protein by the pcDNA *HOXA10 *was decreased compared to the amount expressed by pcDNA *HOXA10*-de-3'UTR which carries the *HOXA10 *3'-UTR deletion, and its effect on suppressing invasion was also less strong. Since miR-135a is expressed endogenously in BT549 cells, it is likely that endogenous miR-135a inhibits *HOXA10 *overexpression by targeting its 3'UTR. Moreover, consistent with invasion assay of BT549 cells transfected with miR-135a inhibitor, overexpression of *HOXA10 *impaired the invasion of BT549 cells.

## Discussion

miRNAs are small noncoding regulatory RNAs that have been studied in various types of cancers. Many miRNAs that regulate epithelial to mesenchymal transition (EMT) [32] and pro-metastatic [5,6] or anti-metastatic functions [9] have been identified. Previously miR-135 has been reported to regulate genes in many other types of cancer [11,14,16,33,34], but its roles in breast cancer was unknown. Our current study provides the first evidence to demonstrate that miR-135a plays a role in promoting migration and invasion of breast cancer cells.

The ability of miR-135a to promote cell migration and invasion was assessed by both over-expression and down-regulation experiments. Remarkably, we observed apparent high levels of miR-135a expression in „triple negative" malignant invasive breast tumors (Figure 1A), and the highly invasive phenotype of the BT549 cell line (Figure 1B), suggesting that miR-135a might play an important role in maintaining metastatic functions. This hypothesis is supported by our experiments showing that inhibition of miR-135a activity impaired the invasion and migration of BT549 cells *in vitro *(Figure 2A). To further verify, this relationship up-regulation experiments were performed in cell lines with different invasive phenotypes. In addition to the enhanced migration and invasive ability of SKBr3 cells induced by increased miR-135a expression (Figure 2B), we also observed enhanced invasive ability of miR-135a-transfected MDA-MB-231 cells (Figure 2C). Although endogenous miR-135a was not detectable in MDA-MB-231 cells they are highly invasive, and we speculate that migration and invasion may not be miR-135a-dependent processes in those cells due to genetic differences between different breast cancer cell lines. Furthermore, miR-135a could not affect the low invasive property of MCF-7 cells even though it was forced overexpressed and led to decreased endogenous HOXA10 protein expression in those cells (Figure 4D). We speculate this is also due to genetic cell specificity. For example, E-cadherin is highly expressed in MCF-7, so we believe that the effect of miR-135a over-expression is not strong enough to induce a change of the migration status of the MCF-7 cells. This suggests that the native non-metastatic character of MCF-7 may have other miR-135a-independent mechanisms that are responsible to maintain the non-aggressiveness of this particular cell type. It is also possible that HOXA10 3'UTR is mutated and cannot be targeted by miR-135a in MCF-7 cells. Future studies are required to define these two possibilities. Indeed it has been reported that HOXA10 is not regulated by miR-135a in MCF-7 cells [14] which is in contrast to our results for other cancer cell lines tested. Unlike the migration and invasion phenotypes, up- or down-regulation of miR-135a did not affect cell proliferation (Figure 2D).

Several HOX genes are regulated by miRNAs [6,19-21]. This work is the first to implicate miR-135a down-regulation of *HOXA10 *expression in breast cancer cell invasiveness. The mechanism by miR-135a targets *HOXA10 *for repression was verified by in vitro 3'-UTR luciferase assays. *HOXA10 *over-expression in miR-135a expressing cells was dependent on the absence of the *HOXA10 *3'-UTR (Figure 3C-D) supporting our conclusion that miR-135a inhibits *HOXA10 *via targeting its 3'-UTR. Our results showed that endogenous *HOXA10 *in MDA-MB-231 was not detectable (Figure 3B) and this could be attributable to methylation of the *HOXA10 *promoter as previously reported [35]. Combined with the fact that individual miRNAs have the potential to modulate the expression of many mRNAs, our result showing miR-135a expression in MDA-MB-231 cells that increased migration and invasion (Figure 2C) suggest there may be miR-135a targets other than *HOXA10 *that can promote migration/invasion events. Indeed, miR135a was reported to be up-regulated in portal vein tumor thrombus and these cells showed increased migration and invasion in vitro. However, the metastasis suppressor 1 gene and not *HOXA10 *was found to be the direct, functional target of miR-135a in this tissue [16]. We propose that this gene may function in some breast cancers to suppress migration and invasion rather than *HOXA10*, and we intend to test this in the near future. Our results also suggested that miR-135a post-transcriptional down-regulation of the *HOXA10 *target gene was not restricted to translation repression (Figure 4A-B, 4D-E) but also occurred by inducing mRNA degradation (Figure 4C), which agrees with previous reports on the action of miRNAs and highly homologous targets [36].

To further investigate the effect of *HOXA10 *on breast cancer cell invasiveness, we overexpressed *HOXA10 *in already highly invasive breast cancer cell lines. Overexpression of *HOXA10 *without 3' UTR targeting by miR-135a led to decreased invasion of MDA-MB-231 cells (Figure 5A), and significantly inhibited the miR-135a-regulated invasion of BT549 cells (Figure 5D). Knockdown of *HOXA10 *increased invasiveness of BT549 cells and could partially rescue the decreased invasive property caused by 135a inhibitor (Figure 5B). Other members in the same gene family have also been shown to play roles in breast cancer. It has previously been reported that HOXA9, a paralog of *HOXA10*, is a tumor suppressor in breast cancer [37], and expression of HOXD10 in MDA-MB-231 significantly impaired migration [38]. Interestingly, in BT549 cells the expression of a full-length *HOXA10 *cDNA was repressed. It is highly likely that the high endogenous miR-135a level inhibits *HOXA10 *expression through targeting its 3'-UTR, since deletion of 3'-UTR led to a higher expression of *HOXA10 *protein than the full-length cDNA *HOXA10 *(Figure 5C), and the effect of *HOXA10 *overexpression on cell invasion varied depending on the absence or presence of the *HOXA10 *3'-UTR (Figure 5D). These results illustrate that miR-135a promoted cell migration and invasion, at least partially, through repression of *HOXA10 *via its 3'-UTR and also verified the in vitro luciferase assay results (Figure 3C-D). However, there is no evidence that miR-135a regulation of *HOXA10 *is exclusive. *HOXA10 *may also be targeted by other miRNAs and it appears that promoter methylation is also an important regulatory mechanism for *HOXA10 *in some tissues [35,39]. Our result is in an agreement with a former study that reported *HOXA10 *expression inhibited matrigel invasion by breast cancer cells [30]. The study also reported that *HOXA10 *expression induced p53 production. Therefore, understanding the molecular mechanism of the regulation of *HOXA10 *by miR-135a may provide a method to explore upstream regulation of *HOXA10 *and connect it with p53 tumor suppressor signalling pathways in breast cancer. Also, the "triple negative" invasive breast cancer type has a very poor prognosis and as yet no antibody target has been reported for the treatment of this type of breast cancer. So we believe future investigation on *in vivo *studies as well as on clinical specimens will confirm the importance of miR-135 and identify additional markers for diagnosis and treatment.

## Conclusions

Although further work is required to fully understand the mechanism by which miR-135a influences tumor metastasis *in vivo*, the identification of miR-135a as a regulator of tumor migration and invasion *in vitro *suggests it may play an important role in mediating the oncogenesis of breast cancer. Our results indicate that miR-135a levels can regulate breast cancer cell migration and invasion, but not proliferation, at least partly through 3'-UTR targeting and repression of *HOXA10 *expression. Furthermore, given the apparent high levels of miR-135a in the „triple negative" malignant metastatic breast cancers, inhibitory targeting of miR-135a may have therapeutic potential for controlling or preventing breast tumor metastasis.

## Competing interests

The authors declare that they have no competing interests.

## Authors' contributions

YC carried out the experimental studies, drafted and completed the manuscript. JZ set up the method called Poly (A) RT PCR for miRNA detection and participated in the design of the study. HW was in charge of the clinical samples selection and performed the proofreading. ZJ, CX, YD and LX disposed the tissue samples. FZ and JZ completed sample conservation. RL refined the manuscript. HZ and DM conceived of the study and performed the statistical analysis. All authors read and approved the final manuscript.

## Pre-publication history

The pre-publication history for this paper can be accessed here:

http://www.biomedcentral.com/1471-2407/12/111/prepub
